# 0.5-keV Soft X-ray attosecond continua

**DOI:** 10.1038/ncomms11493

**Published:** 2016-05-11

**Authors:** S. M. Teichmann, F. Silva, S. L. Cousin, M. Hemmer, J. Biegert

**Affiliations:** 1 ICFO-Institut de Ciencies Fotoniques, The Barcelona Institute of Science and Technology, 08860 Castelldefels, Barcelona, Spain; 2 ICREA-Institució Catalana de Recerca i Estudis Avançats, 08010 Barcelona, Spain

## Abstract

Attosecond light pulses in the extreme ultraviolet have drawn a great deal of attention due to their ability to interrogate electronic dynamics in real time. Nevertheless, to follow charge dynamics and excitations in materials, element selectivity is a prerequisite, which demands such pulses in the soft X-ray region, above 200 eV, to simultaneously cover several fundamental absorption edges of the constituents of the materials. Here, we experimentally demonstrate the exploitation of a transient phase matching regime to generate carrier envelope controlled soft X-ray supercontinua with pulse energies up to 2.9±0.1 pJ and a flux of (7.3±0.1) × 10^7^ photons per second across the entire water window and attosecond pulses with 13 as transform limit. Our results herald attosecond science at the fundamental absorption edges of matter by bridging the gap between ultrafast temporal resolution and element specific probing.

## Abstract (web summary)

Attosecond soft X-ray pulses hold promise for probing electronic dynamics in real time, but it is challenging to achieve element sensitivity while maintaining temporal resolution. Teichmann *et al*. report the cover of carbon, nitrogen and oxygen absorption edges with an isolated pulse supporting 13 as duration.

## Graphical abstract

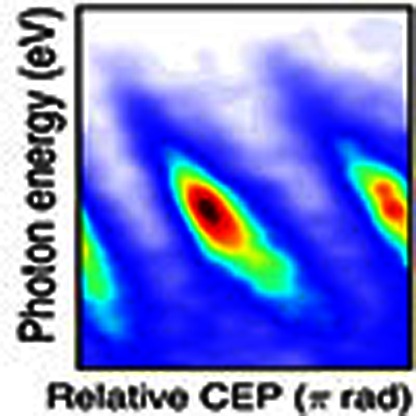

The availability of waveform-controlled pulsed coherent radiation covering the entire water window represents a current frontier in the development of ultrafast soft X-ray sources since this spectral range contains the K-shell absorption edges of carbon (284 eV), nitrogen (410 eV) and oxygen (543 eV) as well as the L-shell absorption edges of calcium (341 eV), scandium (395 eV), titanium (456 eV) and vanadium (511 eV). Controlled coherent radiation consisting of isolated attosecond pulses in this photon energy region would herald a new era of attosecond physics since it would combine its unprecedented temporal resolution with the ability to localize the observed dynamics at a fundamental absorption edge, that is, at a specific atom or site inside a material and follow it to another atom or site. Such attosecond soft X-ray spectroscopy would offer site-specific probes for observing electron correlation^1^,^2^,^3^,^4^ and many-body effects of core-excited atoms^5^,^6^ or attosecond electron transfer^7^ in photo- and electro-chemical processes of organic solar cells and molecular electronics. This would permit investigating semiconductor surfaces where charge transfer occurs on a timescale shorter than 3 fs (ref. 8), and to study insulator-to-metal transitions at the oxygen edge^9^. With water window covering soft X-ray radiation, coherent imaging measurements such as transmissive, reflective and ptychographic diffractive imaging can be envisaged that offer the possibility to resolve structural dynamics of biomolecules at high resolution and fast timescales.

Attosecond light pulses^10^ are generated through coherent frequency upconversion of intense laser pulses via high harmonic generation (HHG), a process that relies on sub-cycle electron recollision in the laser field and recombinative light emission. These dynamics occur during a fraction of the driving laser electric field period and naturally lead to the emission of attosecond or femtosecond bursts of radiation. Contributions from each wave-cycle within a long laser pulse result in the generation of trains of such pulses. On the other hand, limiting the recombination to a single wave-cycle^11^ by using a carrier to envelope phase (CEP) stable sub-2-cycle laser field results in single attosecond pulse emission^11^,^12^,^13^,^14^. High photon energies are achieved through ponderomotive scaling by driving the HHG process with long wavelength lasers^15^,^16^,^17^,^18^,^19^,^20^. With such approach, first CEP-dependent HHG spectra were demonstrated whose cutoff reached 300 eV (refs 18, 19). We have recently validated the viability of such a HHG-based source for soft X-ray absorption fine structure measurement at the carbon K-edge from a solid material^19^, and we have shown that isolated attosecond structures with duration below 355 as can be produced at 300 eV (ref. 20).

Here, we address the to-date unsolved challenge of phase matching CEP controlled HHG with coverage from 200 eV across the entire water window up to the oxygen K-shell edge at 543 eV. Such an achievement is markedly different from coverage of a single absorption edge at 300 eV since only the simultaneous measurement at several absorption edges will permit following an excitation or charge density across the various constituents of a material. For instance, coverage of the chlorine, carbon and nitrogen edges simultaneously, will permit following the formation of an exciton at the chlorine (205 eV) site of the antenna complex of an organic solar cell and its diffusion towards the charge separating interface across the cell's hydrocarbon backbone. We demonstrate experimentally, and investigate theoretically, the regime of high-flux phase matching of soft X-ray continua with CEP control and coverage of the entire water window. Our source now enables interrogation of the most fundamental absorption edges within the water window simultaneously and with attosecond temporal resolution.

## Results

### Phase-matched water window soft X-ray emission

**Figure 1:**
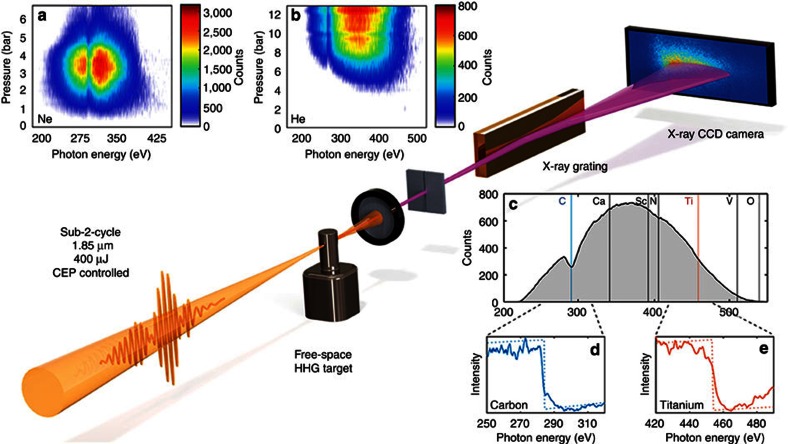
Experimental setup showing pressure dependence and spectral coverage. Top: pressure scans in Ne (**a**) and He (**b**). Clearly visible is the C K-shell absorption edge due to hydrocarbon residue in the beamline. Middle: experimental setup consisting of a high-pressure effusive target, a free-standing IR filter, and the home-built spectrograph that consists of a 2,400 lines per mm gold coated grating and a cooled X-ray CCD. Bottom right: (**c**) a spectrum from HHG in He (2 min integration time) with the reachable K-shell absorption edges indicated by solid vertical lines and L-shell absorption edges indicated by dashed vertical lines. The two graphs below show absorption measurements using foils of 200 nm of carbon (**d**) and titanium (**e**) where the K-edge at 284 eV and L_2,3_-edges at 456 eV, are clearly evident.

A schematic of our setup is shown in Fig. 1. Carrier envelope phase (CEP) stable (see Supplementary Information for details), 1.9-cycle pulses at 1,850 nm wavelength at a repetition rate of 1 kHz are focused with a 100 mm focal length spherical mirror to a peak intensity of 0.5 PW cm^−2^ to generate high harmonic radiation. The HHG target consists of a tube with 1.5 mm outer diameter with 0.3 mm entrance and exit holes through which the laser beam is focused; see the Methods section for further details. A differential pumping scheme allows us to sustain 6 bar of backing pressure with helium while maintaining an ambient pressure of 20 mbar in the generation chamber. Measurements up to 12.5 bar in helium are possible but cannot be sustained for a long time with our present vacuum system. High harmonic radiation is spectrally resolved with a home-built spectrograph consisting of a motorized slit, a flat-field imaging reflective grating (Hitachi, 2,400 lines per mm) and a cooled, back-illuminated soft X-ray charge-coupled device camera (PIXIS-XO-2048B, Princeton Instruments). For a first study, we varied the target backing pressure for neon (Fig. 1a) which gave an optimum yield at 3.5 bar and cutoff at 350 eV, amply covering the carbon K-shell edge at 284 eV. The strongest emission in helium coincided with the highest cutoff at 550 eV, which was obtained at the maximum backing pressure of 12.5 bar (Fig. 1b). This indicates that higher pressures need to be achieved than available in our setup to assess the exact pressure dependence and possible even stronger harmonic signal. Having determined the optimum pressure conditions, we opted to use helium at the maximum sustainable pressure of 6 bar. Figure 1c displays the measured sub-2-cycle-driven soft X-ray continuum, on a linear scale, covering the entire water window up to the oxygen edge at 543 eV. Clearly resolved are the soft X-ray absorption K-shell edges resulting from the insertion of 200 nm thin films of carbon (Fig. 1d) and titanium (Fig. 1e) which are visible at 284 and 456 eV, respectively, when integrating for 2 min. An important parameter in HHG is the position of the generation target relative to the focal position as it is known to sensitively influence phase matching and the contribution of the various quantum paths to harmonic emission^21^. Here, we observe a different behaviour to the known trends for the conditions of maximum yield and highest cutoff for both target gases that permit reaching the water window, that is, for neon and helium. We do not find any significant variation of the cutoff yield when scanning the HHG target within the Rayleigh length, that is, for our conditions within 1 mm on either side of the focal plane. Scanning slightly beyond the Rayleigh length, we observe an immediate loss of signal. This is markedly different from the known smoother transition, and slow decrease of signal, for low-pressure phase matching and 800 nm wavelength^21^,^22^,^23^ and will be discussed later.

### Soft X-ray yield and CEP controlled spectra

**Figure 2:**
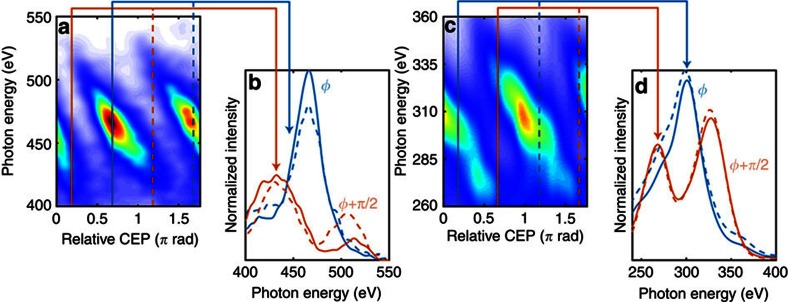
CEP controlled soft X-ray emission and its spectral influence. Shown in **a**,**c** are spectra generated from helium (6 bar) and neon (3.5 bar), respectively, which were acquired for varying CEP in steps of 90 mrad and with 30 s integration time each. The solid lines in **b**,**d** show the dramatic change of the spectral amplitude for two different CEP values; these values are indicated by the coloured vertical lines in **a**,**c**. The excellent CEP stability of the system results in clearly resolved spectra which repeat—as expected—with *π* rad periodicity. This is shown by the dashed lines which are acquired for a *π* rad CEP offset compared with the matching solid coloured lines—see **b**,**d**.

The photon yield and spectral stability of a source are essential for its applications. Having observed the peculiar phase matching dependence on the focusing and target conditions, we investigate its influence on the usability of such HHG soft X-ray source. Such investigation is important since the photon yield needs to be sufficient to permit discrimination of the measurement signal from the noise, and the stability and reproducibility of the spectrum are essential for spectroscopic applications. Hence, we investigate the CEP control and reproducibility of the soft X-ray spectra. Figure 2 shows the spectra measured for a range of CEP values in steps of 90 mrad and for 30 s integration. The clear discrimination between the spectra (Fig. 2a,c) and their reproducibility for an offset of *π* rad (Fig. 2b,d) demonstrates the stability and utility of our soft X-ray source. The importance of CEP control for spectroscopic measurements with ultrafast time resolution is underlined in Fig. 2b,d which exhibit a strong variation of the spectral envelope, and shift of the cutoff, for two different CEP values in neon and helium. The observed spectral changes, and cutoff shifts of up to 10%, of the cutoff energy would have detrimental influence on the utility of harmonic sources for spectroscopic measurements without the demonstrated control and stability. Corresponding photon fluxes of the source at 284 eV are (2.8±0.1) × 10^7^ photons/s/10% bandwidth (BW) in neon and (1.8±0.1) × 10^6^ photons/s/10% BW in helium, resulting in pulse energies, defined only in the water window, between the carbon K-shell edge and the cutoff, of 2.9±0.1 pJ in neon and 0.9±0.2 pJ in helium. Clearly, these energies are sufficient for soft X-ray absorption spectroscopy as shown by the measurements in Fig. 1d,e.

It is interesting to contrast our results with previous work. Our water window flux of (7.3±0.1) × 10^7^ photons/s from neon is similar to the previous reported record value of 6 × 10^7^ photons/s which was however achieved for a non-CEP-stable source and a multi-cycle (40 fs, or >6 cycles) pulse at 2 μm (ref. 24)—note that those driving laser pulses did not result in reproducible isolated attosecond pulse emission. While the achieved general parameters may look similar at first glance, the decisive difference is that we have achieved unprecedented flux for a sub-2-cycle driven CEP controlled soft X-ray continuum which corresponds to a reproducible single attosecond pulse^25^. The measured soft X-ray continua support extremely short durations of 18 and 13 as in neon and helium, respectively. Moreover, driving soft X-ray continua and attosecond generation with a sub-2-cycle pulse is indeed preferable as we have recently demonstrated that a high level of ionization, such as for the conditions of high-pressure phase matching and long wavelengths, has an adverse effect on spectral stability and it could prevent reproducible attosecond pulse generation altogether^26^. Alternative approaches to generate spectral continua, and isolated attosecond pulses, from multi-cycle driving pulses rely on some form of temporal confinement of recollision such that it effectively occurs only once^11^,^27^,^28^. These implementations work very well for 800 nm driven HHG, but they are severely limited for long wavelength-driven HHG due to the required much higher phase matching pressures and the consequently high ionization levels. This limitation is evident from ref. 29 in which ionization gating was implemented for long wavelength (2 μm) -driven multi-cycle HHG. The conditions to achieve a spectral continuum dictated a much lower pressure of 600 torr (0.7 bar) which consequently limited the phase matchable cutoff to 175 eV—far below the water window. This result points at possible severe limitations in implementing the well established gating concepts to achieve attosecond radiation in the water window soft X-ray range. Our implementation circumvents such problems and it is therefore interesting to investigate the conditions of phase matching.

### Transient phase matching of soft X-ray harmonic radiation

**Figure 3:**
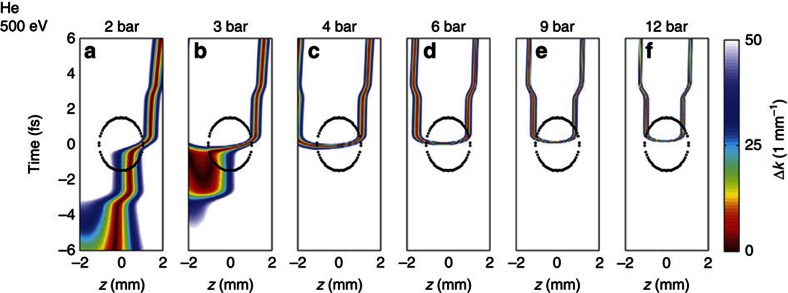
Spatio-temporal phase matching maps as function of target pressure in He. Calculated on-axis phase mismatch as a function of propagation position and time within the pulse for 500 eV radiation generated in helium for our experimental conditions and target pressures of 2–12 bar (**a**–**f**; data for neon can be found in the Supplementary Information). Dark red indicates good phase matching and the back dotted oval area encloses the *z*–*t* space in which the field strength is sufficient to generate 500 eV radiation. At low pressure, phase matching occurs across the entire *z*–*t* range. At high pressure, good phase matching occurs only transiently within a narrow temporal window but across the entire Rayleigh length (here similar to the encircled spatial range).

Having demonstrated the stability and reproducibility of the soft X-ray spectra, we further investigate the peculiar phase matching dependence at high pressure of long wavelength-driven soft X-ray radiation by resorting to numerical simulations. A theoretical investigation is warranted, since the dependence of HHG at these high pressures is not readily investigated experimentally because the harmonic yield varies only moderately within the accessible pressure range, and it is stagnant with target position within the Rayleigh range, but it immediately drops below detection limits outside its phase matching region. Due to the high pressures required for long wavelength-driven HHG, ionization is clearly a major determining factor for phase matching^29^. We therefore investigate the phase mismatch, analogue to ref. 22, for the full range of reachable target pressures as function of time in the laser pulse, and as function of position relative to the focal plane. We choose to illustrate the results of the investigation on the example of highest reachable photon energy of 500 eV in He. The identical behaviour is found for cutoffs of 300 and 400 eV in He and for 300 and 375 eV in Ne—these figures are included in the Supplementary Information. Figure 3 displays the resulting phase matching maps, that is, the on-axis phase mismatch Δ*k*(*t*,*z*), for the high energy end of the soft X-ray spectrum at 500 eV and for the full range of pressures in He. It is important to realize that the phase matching maps alone do not discriminate towards the ability to generate harmonics. Using the cutoff law for HHG, we can however determine the minimum pulse intensity needed to generate 500 eV photons which permits identification of the spatio-temporal region within the phase matching maps where the 500 eV photons are indeed generated. This region is indicated by the black encircled areas in Fig. 3, and the combined conditions evidence phase-matched generation of 500 eV radiation. We find that, at low target pressures (Fig. 3a), phase matching is achieved for positions behind the focal plane (*z*>0 mm), but is limited to a relatively short range of about 0.5 mm and a temporal region before the crest of the pulse. Increasing target pressure, we find a transition regime (Fig. 3b) in which the area of best-phase matching moves to a region before the focus but still occurs temporally before reaching the crest of the pulse. Considering a range of photon energies, that is, not only 500 eV, this regime indicates emission from multiple half-cycles and consequently emission of multiple attosecond bursts. The situation changes markedly at even higher target pressures (Fig. 3c) where optimal phase matching is temporally confined to the centre of the pulse and for an interaction region symmetrically around the focal plane within the Rayleigh range (1 mm on either side of the focal plane). This region persists with even higher pressure (4—12 bar; see Supplementary Discussion on phase matching in focus), and continues narrowing temporally (Fig. 3d–f). We find a trend towards highest pressure (12 bar in Fig. 3f), which exhibits minimization of the temporal spread of the phase-matched region and a symmetric distribution about the focal plane and within the phase matchable region. We note that the condition of minimal temporal spread and symmetric phase matching range coincides with the pressure (12 bar in He) for which we found the strongest harmonic signal experimentally (Fig. 1b). The transition from phase matching at low-pressure downstream from the focal plane to high-pressure phase matching at, and around, the focal plane is explained by the interplay of the various phase matching terms. At low pressure, the interplay between dipole and geometric (Gouy) phase dominate phase matching while the neutral dispersion term is constant as a function of position and electron dispersion is negligible. This results in the well known conditions for best-phase matching downstream from the focal plane^22^. In contrast, at high gas pressure, dispersion from electrons is dominant (see Supplementary Fig. 2) consequently resulting in a symmetric phase matching region around the focal plane because of the spatially symmetric electron dispersion. Repeating the identical analysis for He and for photon energies of 300 and 400 eV, we find the same behaviour; see Supplementary Figs 3 and 4 (Supplementary Figs 5 and 6 for Ne). We also find from our simulation for a pressure of 6 bar in He, that the narrow temporal range coincides nearly identically for the vast range of photon energies of 300, 400 and 500 eV (Supplementary Fig. 7). These findings confirm the existence of a very narrow temporal phase matching window which provides the condition for emission of a single attosecond burst of soft X-ray radiation corresponding to the measured soft X-ray continuum. The lack of interference in the spectral measurements^25^ shown in Fig. 2 further substantiates our conclusion. We note that the existence of such a window for high-pressure phase matching was recently discussed^29^, but was not proven due the lack of CEP control which is known to result in indistinguishability of a attosecond pulse continuum over random spectral shifts^30^. Moreover, the absence of CEP stability would result in random temporal shifts about the cycle duration (∼6 fs for a 2,000 nm pulse) which would inhibit attosecond resolution pump probe measurements. Here, we provide the first proof of these novel phase matching conditions through the demonstrated persistence and repeatability of the soft X-ray continuum and for varying CEP values (Fig. 2).

Our intuitive analysis suggests phase matching conditions resulting in temporal filtering of the macroscopic high harmonic signal and consequently in the reproducible emission of a single attosecond burst. Our findings are in excellent agreement with the experimentally observed parameters and it is this condition that we exploit to generate the high-flux soft X-ray continuum shown in Fig. 2.

## Discussion

We demonstrate the exploitation of a new phase matching regime which allows us to generate the first reproducible and CEP stable attosecond soft X-ray continuum covering the entire water window from 200 to 550 eV. We identify different and unexpected behaviour of harmonic phase matching at the long wavelength-driven high-pressure regime compared with the commonly known conditions at 800 nm and discuss its implications on the stability and reproducibility of attosecond pulse generation. Having identified the crucial importance of CEP control we use the novel conditions to validate our findings by experimentally demonstrating unprecedentedly high flux of CEP controlled and repeatable attosecond continua covering the water window entirely. Our theoretical investigation indicates that the detected continuum is the result of a transient phase matching mechanism resulting in the generation of isolated broadband attosecond bursts. The simultaneous coverage of several absorption edges in combination with attosecond time resolution now enables following excitations and charge migration from one element to another. Thus we have bridged the gap between ultrafast time resolution and element specific probing. These results present a significant step towards studying the entire dynamics of the building blocks of biological life, within organic semiconductors, light harvesting devices and for molecular electronics.

## Methods

### Experiment

The experiment is driven by CEP stable 12 fs, sub-mJ, 1,850 nm pulses at 1 kHz (ref. 19). These pulses are derived from the 7 mJ, 40 fs output of a Ti:Sapphire system after frequency conversion in an optical parametric amplifier^31^, spectral broadening through nonlinear propagation in a hollow core fibre filled with 1.5 bar of argon, and chirp compensation with bulk material^18^. Soft X-rays are generated by focusing the 12 fs pulses into a 1.5 mm long effusive gas target at peak intensities up to 0.5 PW cm^−2^. The radiation is analysed, after removing any remaining fundamental radiation with a 100 nm free-standing aluminium foil, with a home-built spectrograph, consisting of a flat-field reflective grating (Hitachi, 2,400 lines per mm) and a cooled, back-illuminated CCD (Princeton Instruments)^19^.

### Simulations

The phase matching calculations presented in Fig. 3 follow ref. 22. The on-axis phase mismatch between the source and the propagating X-rays is determined for a photon energy of 500 eV considering dispersion of both wavelengths from neutral gas and free electrons, the geometric phase (Gouy phase) of the fundamental as well as the dipole phase of the short trajectories. We do not consider long trajectories since they are effectively suppressed during propagation due to their non-collinear off-axis emission and filtering on pumping apertures of our beamline. We use a Gaussian focus with a waist of 54 μm for the calculations and find the time-dependent fraction of free electrons for a peak intensity of 0.49 PW cm^−2^.

## Additional information

**How to cite this article:** Teichmann, S. M. *et al*. 0.5-keV Soft X-ray attosecond continua. *Nat. Commun.* 7:11493 doi: 10.1038/ncomms11493 (2016).

## Supplementary Material

Supplementary Information Supplementary Figures 1-7, Supplementary Discussion and Supplementary References
