# Supplementary material for: 0.5-keV Soft X-ray attosecond continua
Source: Nat Commun. 2016 May 11;7:11493. doi: 10.1038/ncomms11493 (PMC4865833; doi:10.1038/ncomms11493)
Supplement: Supplementary Information — Supplementary Figures 1-7, Supplementary Discussion and Supplementary References [file ncomms11493-s1.pdf]

Supplementary Figures

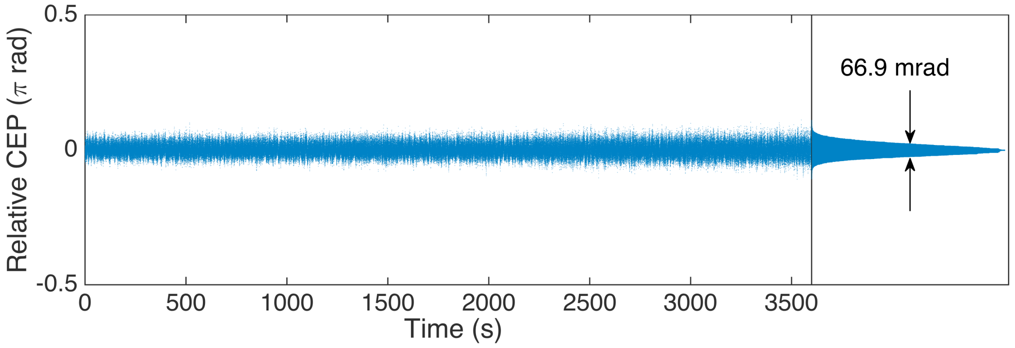

Supplementary Figure 1: Long term CEP stability. F-2f measurement of the 1.85 μm sub-2-cycle pulses for the passively stabilized system. The passive OPA CEP stability of 315.5 mrad over 6 minutes increases markedly with a slow loop. We measure a stability of 66.9 mrad over 1 hour.

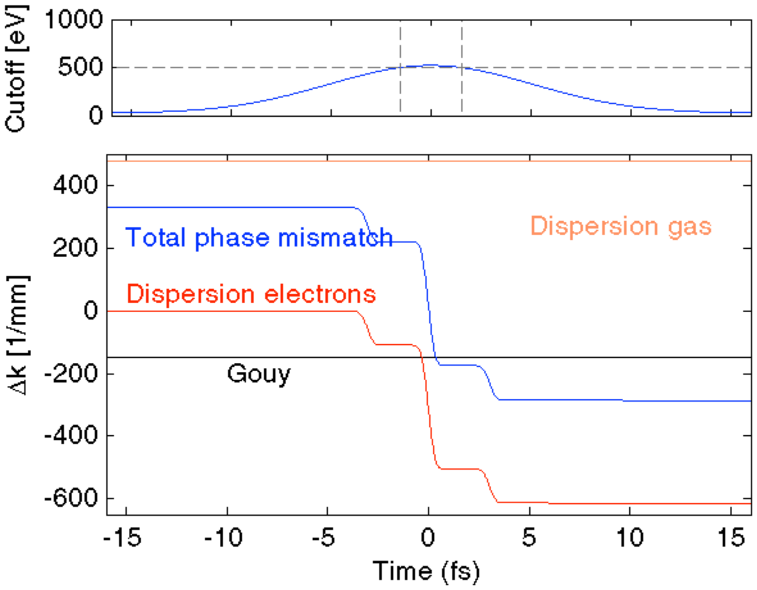

Supplementary Figure 2: Time-dependence of phase mismatch contributions. Calculations for z = 0 mm (in focus) and a target pressure of 6 bar (see Fig. 3d main text). Top panel: Cutoff energy of the emitted radiation. The dotted lines indicate in which temporal range the intensity is sufficiently high for the emission of 500 eV photons. Bottom panel: Phase mismatch contributions showing the strong temporal dependence of the phase mismatch (blue) mediated by the free electrons (red).

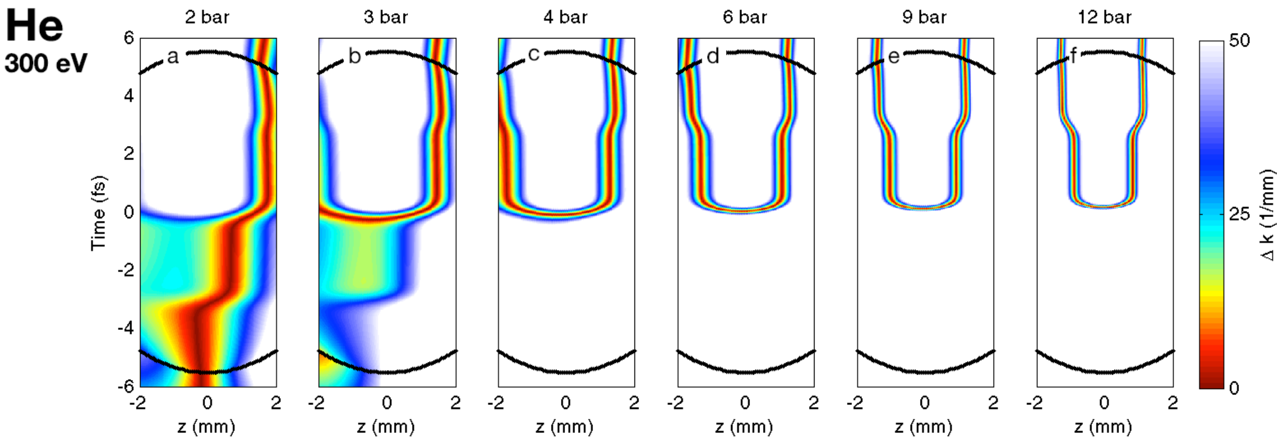

Supplementary Figure 3: Spatio-temporal phase matching maps as function of target pressure in He (300 eV). Calculated on-axis phase mismatch as a function of propagation position and time within the pulse for 300 eV radiation generated in helium for our experimental conditions and target pressures of 2 to 12 bar. Dark red indicates good phase matching and the back dotted oval area encloses the z-t space in which the field strength is sufficient to generate 300 eV radiation. At low pressure, phase matching occurs across the entire z-t range. At high pressure, good phase matching occurs only transiently within a narrow temporal window but across the entire Rayleigh length.

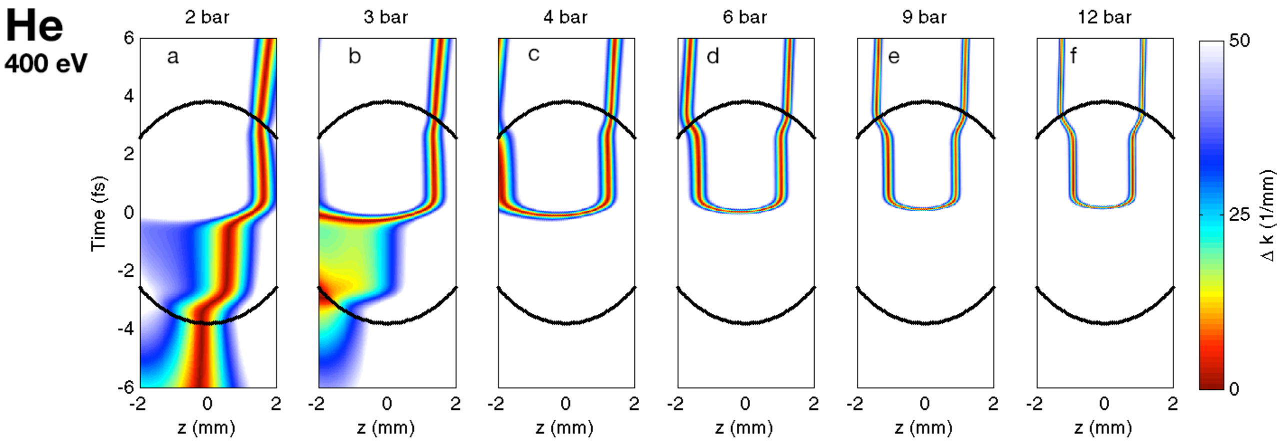

Supplementary Figure 4: Spatio-temporal phase matching maps as function of target pressure in He (400 eV). Calculated on-axis phase mismatch as a function of propagation position and time within the pulse for 400 eV radiation generated in helium for our experimental conditions and target pressures of 2 to 12 bar. Dark red indicates good phase matching and the back dotted oval area encloses the z-t space in which the field strength is sufficient to generate 400 eV radiation. At low pressure, phase matching occurs across the entire z-t range. At high pressure, good phase matching occurs only transiently within a narrow temporal window but across the entire Rayleigh length.

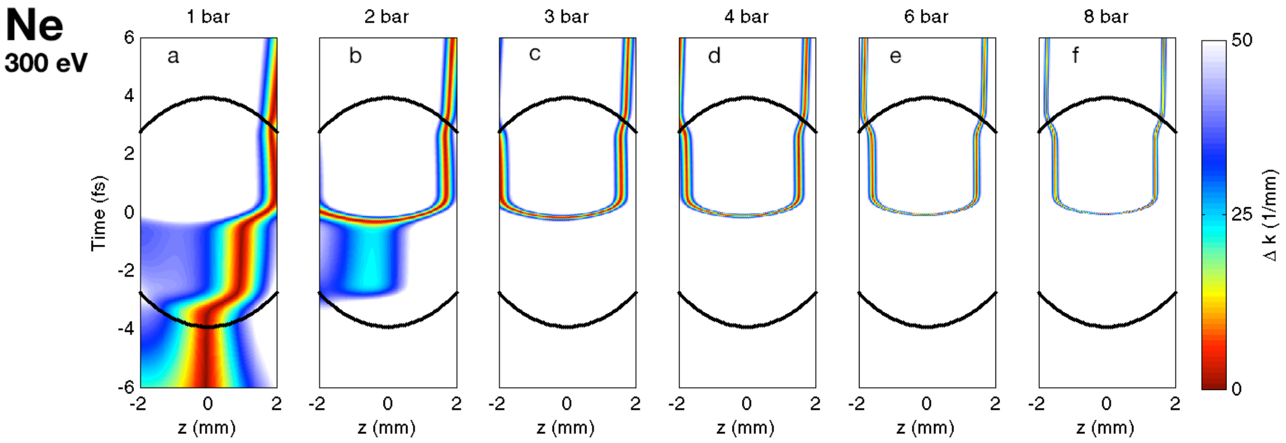

Supplementary Figure 5: Spatio-temporal phase matching maps as function of target pressure in Ne (300 eV). Calculated on-axis phase mismatch as a function of propagation position and time within the pulse for 300 eV radiation generated in neon for our experimental conditions and target pressures of 2 to 8 bar. Dark red indicates good phase matching and the back dotted oval area encloses the z-t space in which the field strength is sufficient to generate 300 eV radiation. At low pressure, phase matching occurs across the entire z-t range. At high pressure, good phase matching occurs only transiently within a narrow temporal window but across the entire Rayleigh length.

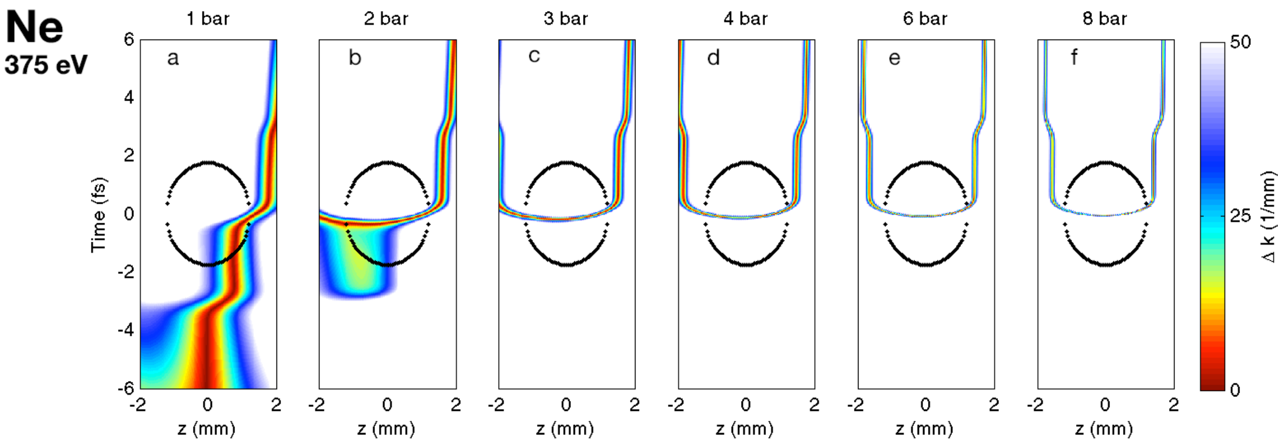

Supplementary Figure 6: Spatio-temporal phase matching maps as function of target pressure in Ne (375 eV). Calculated on-axis phase mismatch as a function of propagation position and time within the pulse for 375 eV radiation generated in neon for our experimental conditions and target pressures of 2 to 8 bar. Dark red indicates good phase matching and the back dotted oval area encloses the z-t space in which the field strength is sufficient to generate 375 eV radiation. At low pressure, phase matching occurs across the entire z-t range. At high pressure, good phase matching occurs only transiently within a narrow temporal window but across the entire Rayleigh length (here similar to the encircled spatial range).

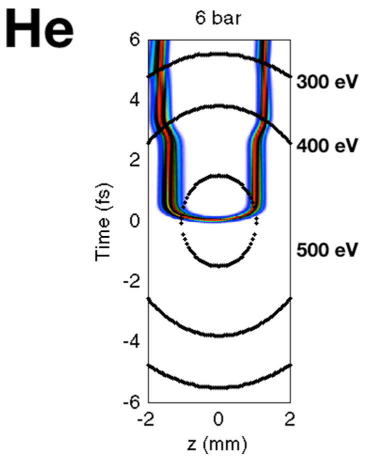

Supplementary Figure 7: Spatio-temporal phase matching maps as function of target pressure in Ne (375 eV). Calculated on-axis phase mismatch as a function of propagation position and time within the pulse for 375 eV radiation generated in neon for our experimental conditions and target pressures of 2 to 8 bar. Dark red indicates good phase matching and the back dotted oval area encloses the z-t space in which the field strength is sufficient to generate 375 eV radiation. At low pressure, phase matching occurs across the entire z-t range. At high pressure, good phase matching occurs only transiently within a narrow temporal window but across the entire Rayleigh length.

Supplementary Discussion

CEP Stabilisation

The Ti:Sapphire system itself is not CEP stabilized, but we make use of the fact that a seeded optical parametric amplifier produces a self-CEP stable idler if pump and signal (seed) are driven by the identical laser pulse [1,2]. The CEP of the idler output is insensitive to the phase fluctuations

of the Ti:Sapphire pulse and simply experiences a fixed but unknown phase offset. A simple time delay between pump and seed can be used to shift the CEP offset to any desired value. Owing to environmental fluctuations, a slow CEP drift can occur which we measure as 315.5 mrad over 6 minutes. These slow CEP fluctuations are monitored by sampling a fraction of the octave-spanning hollow-core fibre output to generate single-shot f-2f interferometric spectra. The CEP fluctuations are then inferred from the processed spectra where after compensation can be introduced via a piezo-electric transducer for a pump delay offset in the OPA. With such slow loop correction, CEP fluctuations have been kept at 88.9 mrad for periods of 3 hours [3] and we measured 67 mrad over 1h – see Supplementary Fig. 1.

Transient Phase matching

The transient phase matching effect discussed in the main text finds its origin in the strong dependence of the phase mismatch on the electron density at high target pressures. To illustrate this, we plot in Supplementary Fig. 2 the temporal dependence of the different phase mismatch contributions in focus (z = 0 mm) for the optimal case of a target pressure of 6 bar (see Fig. 3d in the main text). Dispersion of the neutral gas (orange) and the Gouy phase (black) result in a positive, near-constant offset of the phase mismatch. This is compensated in a very narrow temporal window around the crest of the pulse due to the free electron contribution (red), as shown by the blue curve.

In the following we combine the temporal phase matching (Supplementary Fig. 2) with propagation and show temporal spatial phase matching plots. Supplementary Figures 3 and 4 are calculated for He and identical conditions as for Fig. 3 in the manuscript, but for photon energies of 300 eV and 400 eV. We find identical behavior as for the 500 eV case. Clearly visible is the need for high target pressures to get appreciable broadband phase matching, typically 4 bar and higher, which is in accordance with our experimental findings. For completeness, we show identical plots but for Ne and for a peak intensity of 0.37 PW/cm^2. Similar behavior is found as for He but, as expected for a gas with lower ionization potential, for a phase matching pressure of 3 bar and higher. Supplementary Figure 7 summarizes our findings in Supplementary Figs. 3, 4 and Fig. 3. The plots shows the three calculations for 300 eV, 400 eV and 500 eV superimposed for 6 bar pressure in He. We find broadband phase matching and temporal narrow confinement for all cases with only small deviation as function of photon energy.

In the spatial dimension, the above observations are at odds with the low-pressure regime in which phase matching of short trajectories occurs asymetrically around the focal plane with the optmial target position downstream of the focal plane [4]. The interplay of the dipole and geometric (Gouy) phase is responsible for this condition since the neutral dispersion term is constant as a function of position and electron dispersion is negligible. In the present case of high pressure phase matching, however, the dipole as well as the geometric phase are dominated by dispersion (Supplementary Fig. 2). A symmetric phase matching region around the focal plane, as highlighted in the main text and seen in Figs. 3 and Supplementary Figs. 3-7, is thus a consequence of the likewise spatially symmetric electron dispersion.

Supplementary References

1. Baltuska, A. et al. Controlling the Carrier-Envelope Phase of Ultrashort Light Pulses with Optical Parametric Amplifiers. *Phys. Rev. Lett.* **88**, 133901 (2002).

2. Hauri, C.P. et al Phase-preserving chirped pulse parametric amplification of 17.3 fs pulses from a Ti:Sapphire oscillator. *Opt. Lett.* **29**, 1369-1371 (2004).

3. Cousin, S. L. et al. High-flux table-top soft x-ray source driven by sub-2-cycle, CEP stable, 1.85-µm 1-kHz pulses for carbon K-edge spectroscopy. *Opt. Lett.* **18**, 5383-5386 (2014).

4. Balcou, P. et al. Generalized phase-matching conditions for high harmonics: the role of field-gradient forces. *Phys. Rev. A* **55**, 3204–3210 (1997).
